# A scoping review on sexual and reproductive health behaviors among Tanzanian adolescents

**DOI:** 10.1186/s40985-019-0114-2

**Published:** 2019-09-03

**Authors:** Hamida Nkata, Raquel Teixeira, Henrique Barros

**Affiliations:** 1 EPIUnit – Instituto de Saúde Pública, Universidade do Porto, Rua das Taipas, nº 135, 4050-600 Porto, Portugal; 2 Nkinga Institute of Health and Allied Sciences, P.O. Box 60, Nkinga, Tabora Tanzania; 3 Departamento de Ciências da Saúde Pública e Forenses e Educação Médica, Faculdade de Medicina, Universidade do Porto, Porto, Portugal

**Keywords:** Reproductive health, Sexual behavior, Adolescents, African Eastern

## Abstract

**Background:**

There is wide variation among societies in profiles of adolescent health and behaviors, but they all experience sexual and reproductive health as a major challenge. However, adolescents in middle- and low-income countries are of particular concern, as it is the case in Tanzania, where limited social, educational, and health services contribute to make them victims of unwanted pregnancies, unsafe abortion, and sexually transmitted infections including HIV. Thus, we undertook a descriptive systematic scoping review of the available published information on sexual and reproductive health among Tanzanian adolescents.

**Methods:**

We performed a scoping review to collect and analyze observational data on sexual and reproductive health behaviors among Tanzanian adolescents. Publications were identified using PubMed®, Scopus®, Web of Science™, and Cochrane Library electronic databases from 2000 to December 2017. A protocol was defined to identify relevant studies. We included original observational studies conducted in Tanzania and published in English, both quantitative and qualitative, involving adolescents (10 to 19 years old), and that considered at least one of the following items: condom use, number of sexual partners, sexual debut, contraceptive prevalence, sexually transmitted infections, unwanted pregnancies, abortion, or knowledge about reproductive health. All included articles were coded according to relevant exposures or outcomes and subsequently analyzed to assess frequencies.

**Results:**

After screening for inclusion criteria, 13 publications were included in the datasheet developed to record the findings. Overall, the publications revealed that adolescents tend to be sexually active, with high rates of early sexual debut, have multiple sexual partners, and a limited use of condom and contraceptives. Sexual coercion and transactional sex were also frequent. Only one study addressed pregnancy as an outcome, and a single study looked at the relevant health services. No study was retrieved describing the frequency of unsafe abortion.

**Conclusion:**

Adolescents engage in high-risk sexual behaviors and experience its adverse consequences. It is essential to collect more information, but the existing evidence supports a need for improving provision of sexual and reproductive health services among Tanzanian adolescents.

## Background

Tanzania is a large country located in East Africa, with a total population of 44.9 million as per 2012 census, and has the second youngest population in the region, the median population age being 18 years [1, 2]. The country clearly experiences challenges in adolescent’s sexual and reproductive health since Tanzania is among the ten countries presenting the highest frequency of adolescent pregnancy [3]. By the age of 19, almost half of all girls are pregnant or have given birth to a child [4]. Moreover, it is estimated that 5% of people aged 15 years and older are living with human immunodeficiency virus (HIV) in Tanzania [5].

Despite these challenges, only about one third of health facilities in Tanzania offer youth-friendly services [6], where adolescents get contraceptives, testing for HIV, and treatment for sexually transmitted infections (STI’s). These important services have remained more in theoritical realm with use of contraceptives among adolescents remaining as low as 8.6% and Tanzanian adolescents continuing to experience relevant barriers to access reproductive health information and care [4, 7, 8].

Although in many communities pre-marital sex is culturally or religiously forbidden, several studies evidenced that adolescents practice pre-marital sex [9–12]. Consequently, in the absence of effective prevention, adolescents are exposed to unwanted pregnancies, unsafe abortion, and STIs including HIV. It is believed that sexual behaviors in their individual or community dimensions can be modified and mediate changes in the frequency of multiple adverse outcomes. Thus, it is essential to know and understand the dynamics of sexual behaviors as a first step to plan needed interventions and policy evaluations. This study reviews the available published information on sexual and reproductive health behaviors among Tanzanian adolescents, concerning the years 2000–2017

## Methods

This current scoping review followed a five-stage methodological framework that included (1) identifying the research question and (2) the relevant studies, (3) selecting the studies according to inclusion criteria, (4) charting and interpreting data, and (5) summarizing and reporting of results [13]. Also, we adapted as appropriate the Preferred Reporting Items for Systematic Reviews and Meta-analysis (PRISMA) statement [14], although we did not previously register a protocol. Our research question was broad—what was the type and extent of available information on sexual and reproductive behaviors among Tanzanian adolescents.

### Search strategy

A comprehensive literature search was conducted to identify all relevant studies reporting on sexual and reproductive behaviors of Tanzanian adolescents, by searching four international databases (PubMed®, Scopus®, Web of Science™, and Cochrane Library), for peer-reviewed publications from January 2000 to December 2017. In addition, backward citation tracking on the eligible studies was applied to identify additional sources of information.

The search strategy included a range of relevant combination of keywords: (“sexual health” OR “reproductive health” OR “family planning” OR “sex education” OR “sexual behavior” OR “contraception” OR “contraceptive agents” OR “condoms” OR “pregnancy” OR “abortion” OR “termination of pregnancy” OR “sexually transmitted infections” OR “venereal diseases”) AND (“Adolescent” OR “youth” OR “young people” OR “teenager”) AND Tanzania.

Articles were included if they met the following criteria: (a) original observational studies, both quantitative and qualitative; (b) involve adolescents, 10 to 19 years old; (c) describe at least one of the following: condom use, sexual partners, sexual debut, contraceptive prevalence, sexual transmitted infections, unwanted pregnancies, abortion, or sexual/reproductive health knowledge; and (d) conducted in Tanzania. Only articles in the English language were included. Our search strategy looked for papers published from January 2000 to December 2017 published after the Millennium Development Goals (MDG) declaration [15].

To reduce the potential for reviewer bias, titles and abstracts of all identified records were independently screened by two authors (HN and RT) and checked for agreement. Subsequently, the full text of potentially relevant studies was read and independently screened for the eligibility criteria. Discrepancies in the study selection were resolved by consensus or were discussed with a third author (HB) for a final decision.

**Fig. 1:**
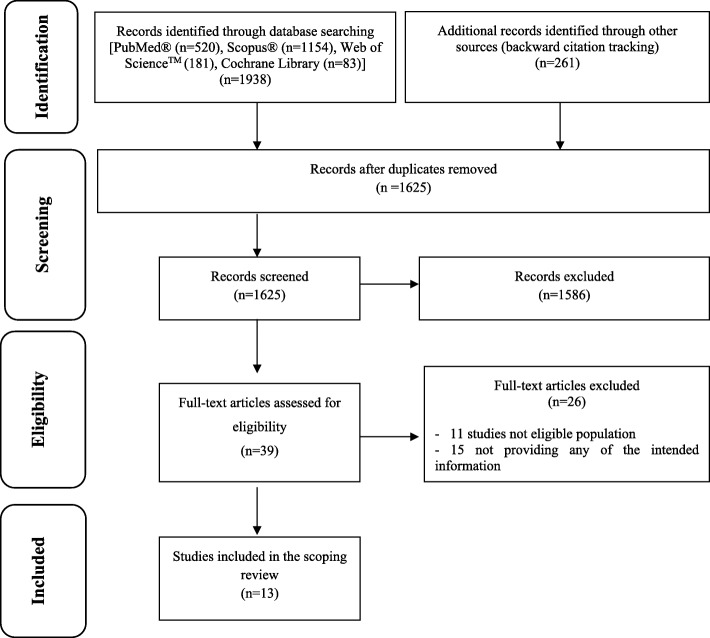
PRISMA flow diagram

In the first step of the literature search, 1938 references were identified. Additional records identified through backward citation counted 261. After screening for title and abstract, 39 documents were selected for further analysis by reading the full text. Finally, 13 studies met inclusion criteria and were selected for data extraction (Fig. 1).

### Charting data and data extraction

Data was extracted from each publication by two reviewers (HN and RT) using a structured data sheet specifically developed by the authors. We extracted the following information from each included study: (1) author, (2) title, (3) publication year, (4) geographical location, (5) study design, (6) sample size, (7) age range of participants, (8) prevalence of condoms use/non-use, (9) prevalence of sexually active adolescents, (10) prevalence of multiple sexual partners, (11) prevalence of sex at or before age 14, (12) prevalence of transactional sex, (13) prevalence of STIs including HIV, (14) barriers to reproductive health services, (15) prevalence of pregnancy, (16) prevalence of adolescents tested for HIV, and (17) prevalence of coercion into sexual intercourse.

## Results

**Fig. 2:**
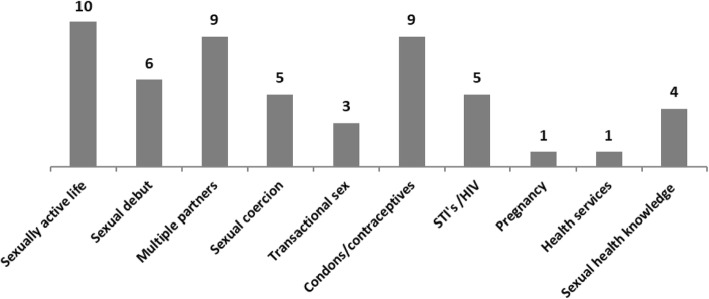
Number of studies addressing the main behaviors, experiences, and outcomes evaluated in the reviewed studies

Of the 13 identified studies, 11 used a quantitative methodology (cross-sectional design), one used a qualitative approach, and one study used both methodologies (Table 1). The sample size ranged from 197 to 9945 participants, with the majority of studies (*n* = 11) including both boys and girls and two focusing on girls. Eight studies took place in school settings [10, 12, 16, 17, 19–22]. Of these, three also examined out-of-school adolescents in their households or villages [17, 19, 20]. Two studies were conducted in health facilities [8, 24], three exclusively in households, and one in rural villages [18, 19, 23]. Figure 2 shows the number of studies addressing the main behaviors and outcomes evaluated in the reviewed studies.

**Table 1:** Studies on sexual and reproductive health among adolescents in Tanzania

Author, year of publication	Geographical location	Study design	Sample size	Study title	Age range (years) or school grade	Summary of findings
1. Kalolo and Kibusi (2015) [10]	Newala, Mtwara	Cross-sectional	403	The influence of perceived behavior control, attitude and empowerment on reported condom use and intention to use condoms among adolescents in rural Tanzania	14–19	- 40.6% prevalence of sexually active participants (57.3% boys, 42.7% girls) - 49.8% had multiple concurrent sexual partners - 15.3% had sex before 14 years of age - 50.6% did not use condom at the last sexual intercourse - 77% indicated they intent to use condoms in the future.
2. Njau et al. (2013) [16]	Rungwe district in Mbeya region	Cross-sectional	324	Correlates of use of condoms among sexually active youth in Southern Highlands, Tanzania	14–18	- 70% prevalence of sexually active participants - 62.5% had multiple concurrent sexual partners - 52.8% prevalence of condom use in the last 3 months - 50.7% of the sexually active adolescents were tested for HIV
3. Mbeba et al. (2012) [8]	Mtwara	Qualitative study	9 focus group (8 to 10 persons^1^ per group)	Barriers to sexual reproductive health services and rights among young people in Mtwara district, Tanzania	10–18	- The age range at sexual debut was 9 to 12 years - Girls reported to not having a place where they can talk about sex, contraception and STIs - Health services were inaccessible due to lack of privacy, confidentiality, equipment and negative attitudes from service providers, such as stigma and discrimination - Girls reported transactional sex (in exchange for money or food) and sexual abuse - Community members have the misconception that contraceptives will harm the fertility of young girls - Community members and service providers think it is inappropriate to girls have access to sexual and reproductive health services and family planning
4. Exavery et al. (2012) [17]	Mpwapwa and Mbeya Rural Districts	Cross-sectional	1327	Acceptability of condom promotion among 10–19 years old adolescents in Mpwapwa and Mbeya Rural Districts, Tanzania	10–19	- 21.8% prevalence of sexually active participants - 38.9% of the boy and 34.7% of the girl participants accept condom promotion and distribution - 18.9% were aware of places where condoms were available or distributed freely - 79.4% agrees with condom effectiveness in preventing transmission of STIs
5. Mmbaga et al. (2012) [12]	Morogoro Municipality	Cross-sectional	316	Incidence and predictors of adolescent’s early sexual debut after three decades of HIV interventions in Tanzania: a time to debut analysis	16–19	- 48.7% prevalence of sexually active participants (52% boys, 48% girls) - 57.8% had sex before 15 years of age - The age range at sexual debut was 8 to 19 years - 13.1% had more than 2 sexual partners in the last 6 months - Prevalence of transactional sex was 28.1% (62.8% girls, 31.8% boys)
6. Mnyika et al. (2012) [18]	Moshi Rural District	Cross-sectional	668	Perceptions of AIDS risk and condom use among out-of-school adolescents in Moshi Rural District, northern Tanzania	10–19	- 45.4% prevalence of sexually active participants (68.1% boys, 31.9% girls) - 70.5% had multiple concurrent sexual partners
7. Exavery et al. (2011) [9]	Four district in Tanzania: Kigoma, Kilombero, Rufiji, and Ulanga	Cross-sectional	612	Multiple sexual partners and condom use among 10–19 year-olds in four districts in Tanzania: What do we learn?	10–19	- 23.4% prevalence of sexually active participants - 42.0% had multiple sexual partners in the last 12 months - 39.2% prevalence of condom use in the last sexual intercourse (41.1% boys, 58.9% girls)
8. Kazaura and Masatu (2009) [19]	Konde, Iringa, and Mara	Cross-sectional	2749	Sexual practices among unmarried adolescents in Tanzania	10–19	- 32.2% prevalence of sexually active participants - 14.8% had multiple concurrent sexual partners - 42% had used condoms at last sexual intercourse - 15.9% had sex unwillingly. Of these, 76% was due to rape (70.3% were girls) - 7.5% reported practicing anal sex
9. Masatu et al. (2009) [20]	Diocese of Evangelical Lutheran Church in Tanzania which included Konde, South-Western, South Central, Iringa, and Diocese in Mara Region	Cross-sectional	2928	Predictors of risky sexual behavior among adolescents in Tanzania	10–19	- 30.6% prevalence of sexually active participants - Mean age of sexual debut was 13.5 years - 24.5% had multiple sexual partners - 47.8% reported use of condoms at last sexual intercourse
10. Kigombola (2006) [21]	Rural Kisarawe	Cross-sectional	334	Knowledge, attitude and practices on HIV/AIDS, its transmission and prevention among primary school pupils in Rural Kisarawe	Sixth and seventh grade students (84% at 13 to 16 years old)	- 41.9% prevalence of sexually active participants - 78.6% sex before or at age 14 - 58% of the girls and 15.7% of the boys were forced into their first sexual intercourse - 28.6% reported use of condoms at last sexual intercourse - 81% had good overall knowledge on HIV/AIDS - 61.1% considered condoms ineffective in preventing HIV - 7.5% previously affected by STI
11. Todd et al. (2004) [22]	Rural Tanzania	Cross-sectional	9283	The sexual health of pupils in years 4 to 6 of primary schools in rural Tanzania	Primary school students approximately 14 years and over	- 37.6% prevalence of sexually active participants (75.2% boys, 24.8% girls) - 72.4% of the boys and 46.2% of the girls reported they had more than 2 lifetime sexual partner - 23.3% of the girls had ever been forced to have sex by a boy or man - 0.1% of the boys and 0.2% of the girls were infected with HIV - 0.3% of the boys and 1.6% of the girls were infected with *Chlamydia trachomatis* - 0.04% of the boys and 0.2% of the girls were infected with *Neisseria gonorrhea* - 0.8% overall prevalence of pregnancy - Prevalence of pregnancy increased from 0.3% in 14 year olds to 2.5% in 17 years and above
12. Obasi et al. (2001) [23]	Rural Mwanza region	Cross-sectional	9445	Prevalence of HIV and Chlamydia trachomatis infection in 15–19-year olds in rural Tanzania	15–19	- 0.6% of the boys and 2.4% of the girls were infected with HIV - Prevalence of HIV increased with age among boys (0.2% in 15 years to 1.0% in 19 years old) and girls (0.9% in 15 years to 4.6% in 19 year old) - 1.0% of the boys and 2.4% of the girls were infected with *Chlamydia trachomatis*
13. Rasch et al. (2000) [24]	Dar-es-Salaam	Cross-sectional	197	Adolescent girls with illegally induced abortion in Dar-es-Salaam: the discrepancy between sexual behavior and lack of access to contraception.	14–19	- 9.3% had sex before or at age 14 - Median age of sexual debut was 16 years - 67.5% had more than 2 sexual partners - Average number of sexual partners was 1.7 - 7.1% contraceptive prevalence - Majority of girls reported to have knowledge of condoms and oral contraceptives - 0.5% previously affected by STI
	Dar-es-Salaam	Qualitative	51			- 41.2% sex before or at age 14 - 13.7% previously affected by STI - Girls reported transactional sex (in exchange for money or gifts) - Many of the girls were forced to have sex, especially in the first sexual intercourse - Girls have a superficial knowledge of modern contraception and are deeply misinformed about its use

^1^Adolescent girls (10 to 18 years), community leaders, and adults (men and women)

The age at first sexual experience was described in three studies and ranged from 8 to 19 years [8, 12, 24], and the prevalence of sexually active participants described in 10 studies varied from 21.8 [17] to 70.0% [16]. Sexual coercion was reported by five studies, and three of them describe its prevalence, which varied from 15.9% [19] to 32.9% [21], girls being significantly more coerced than boys (58% vs. 15.7%) [21].

Three studies reported on adolescents practicing sex for money [8, 12, 24], but only one measured its prevalence, which was 28.1% (62.8% girls vs 31.8% boys) among participants 16 to 19 years old [12]. Nine studies reported adolescents experiencing multiple sexual partners, and the prevalence ranged from 13.1 [12] to 72.4% [22].

One study conducted with girls (14 to 19 years) admitted in a hospital due to induced abortion identified that only 7.1% had ever used a modern contraceptive [24]. The majority of the girls reported knowledge on oral contraceptives, but qualitative interviews revealed superficial knowledge and misinformation about its use and side effects [24]. The prevalence of condom use reported by five studies [9, 16, 19–21] varied from 28.6 [21] to 52.8% [16] while one reported non-use of condoms by 50.6% of the participants [10]. Acceptability of condom promotion and distribution among adolescents was 37% (34.7% girls vs. 38.9% boys), and less than 20% were aware of places where condoms were available or distributed freely [17]. Two studies investigated the knowledge on condom’s effectiveness in preventing HIV, where 79.4% of the participants considered it to be effective [17], and 61.1% to be ineffective [21].

Youth-friendly health service access was investigated by the qualitative study where girls reported not having a place within their communities to visit and talk about sex-related issues [8]. Health services were reported inaccessible due to lack of privacy, confidentiality, equipment, and negative attitudes from service providers, such as stigma and discrimination [8]. Community members, but also health service providers, expressed the misconception that contraceptives will harm the fertility of young girls, and therefore, family planning should not be used by them [8].

The STI prevalence, excluding HIV infection, was described in four studies and ranged from 0.1 to 13.7% among adolescents 12 to 19 years old [21–24]. Two studies reported HIV prevalence between 14 and 19 years which was 0.1% of the boys and 0.2 % of the girls in the first study and 0.6% of the boys and 2.4% of the girls in the second study [22, 23], which also shown that prevalence increased with age among both sexes. However, according to a single study, only 50.7% of the sexually active adolescents ever tested for HIV [16]. Knowledge on HIV was reported by only one study performed with 15 to 16 years old adolescents from a rural area, and 81% had a good overall knowledge on HIV transmission [21]. Prevalence of pregnancy was examined by one study (0.8%) that identified an increasing trend with age (0.3% in 14 years to 2.5% in 17 years and above) [22].

## Discussion

The reviewed studies indicated that Tanzanian adolescents are exposed to high-risk sexual behaviors and consequently to an increased risk of adverse outcomes. Adolescents have multiple sexual partners, early sexual debut, limited use and acceptability of condoms, limited contraceptive use, and misinformation about sexual and reproductive health and are facing sexual coercion and transactional sex. As a result, they are experiencing pregnancy and being infected with common STIs, such as *Chlamydia trachomatis* and *Neisseria gonorrhea*, and also with HIV.

The majority of the studies reviewed reported a worrisome prevalence of early sexual debut. A recent study conducted in South Africa reported that early sexual debut was more frequent among females, living in rural areas and from low-income families [25]. Also, the connection with friends who are engaged in sexual activities and alcohol intake may be connected to early sexual debut [26]. Early sexual debut has been shown to set the path to further engagement in increased sexual risk behaviors and to experience violence [27], is linked to a higher risk of STIs [28] and delinquency experiences compared to those who debut on time [29].

Sexual coercion presented a noteworthy prevalence in the reviewed papers addressing the topic. A study published after our review process was terminated and conducted in Tanzania reported a similar prevalence of sexual coercion among boys and girls (21%) [30]. Our data also stressed the high risk of sex coercion associated with girls, those who usually experience more vulnerable conditions. These findings agree with a previous report from the United Nations Children’s Fund stating that about one in every three adolescent girls has been sexually abused at least one time before the age of 18 [6]. Similarly, transactional sex, defined as sex in exchange for money, food, protection, or shelter, was identified in this review, mostly among girls. The association with poverty and gendered economic inequality, pushing girls to engage in transactional sex at early ages, has been previously described [31]. These results pointed out the need to analyze this problem under the scope of sexual exploitation and abuse of adolescents since it is a clear violation against human rights [32].

Adolescents in Tanzania are experiencing multiple sexual partners which may be associated with limited knowledge of STIs and HIV or prevention activities in sexual and reproductive health [4]. Low average grades at school, frequent alcohol consumption, and low levels of parental monitoring have also been described as factors associated with risk taking among adolescents [33]. The limited use of contraceptives among the adolescents evaluated in the reviewed studies may result from lack of youth-friendly services [7]. Lack of knowledge on modern contraceptives found in our review was also identified in a study conducted in Nigeria with adolescents between 13 and 19 years, where only 5% of the girls reported oral contraceptives as a known family planning method [34]. Yet, adolescents are not aware of their rights and the national policy that provides youth-friendly services [35]. A previous study performed in a STI clinic in Tanzania revealed that condom use among adolescents was less than 50% in community surveys, highlighting the same limited use of condom [35]. This prevalence may be strongly linked to the limited acceptability of condoms promotion and distribution among adolescents identified in our review, as well as to the disregard related to the condom’s effectiveness. The low acceptability referred to the assumption by the adolescents that promotion and distribution of condoms will encourage sexual activity [17].

The prevalence of HIV identified in this review is similar to that described in national population samples of Tanzanian adolescents aged 15 to 19 years that remained 1% in 2007/2008 and 2011/2012 [36]. However, considering barriers experienced in Sub-Saharan Africa to uptake HIV testing [37], data might not reflect the reality since many adolescents have not been tested for HIV. Indeed, this review identified limited percentage of sexually active adolescents who underwent testing for HIV (50.7%).

Review studies provided a 0.8% prevalence of pregnancy among adolescents [22], which is much lower than previously reported (27.1%) [35], reflecting the different settings where the studies were conducted. The study presenting the smaller percentage was conducted within a school environment where pregnant adolescents are expelled immediately after identification of pregnancy [38]. Meanwhile, the one describing large percentage was conducted with adolescents attending a STI clinic in Dar-es-Salaam city, probably reflecting the other extreme of reality. Adolescent pregnancy is a complex public health problem and related to increased prevalence of both maternal and neonatal complications [39]. In Tanzania, neo-natal mortality is much higher among adolescents (41 per 1000) than mothers aged 20 to 29 years (22 per 1000 live births) [40].

Our search strategy looked for papers published from January 2000 to December 2017 to consider the potential impact on Millennium Development Goals (MDG) declared in 2000 [15]. However, one paper described data from 1997 although it was published in 2000 [24]. This review intended to pay special attention to goal number 6(a): to halt by 2015 and begun to reverse the spread of HIV/AIDS. Analysis contextualized on MDGs shows that measures to stop or reverse the spread of HIV among the general population in Tanzania allowed achieving MDG n.6 (a). However, our results indicated that attention should be focused on the adolescent population given the spread of risky sexual behaviors identified. Another relevant point refers to MDG n.3 since gender equality has not been increasing as girls are substantially more exposed to sex coercion and transactional sex and are the ones who carry the burden of an unplanned pregnancy and unsafe abortion. Tanzania did not manage to meet the MDG n.5, which aimed to reduce maternal mortality by three quarters between 1990 and 2015. The target was to reach 133 per 100,000 live births by the year 2015 [41], and if unsafe abortion is not strongly addressed, major threats remain to efficiently decrease maternal mortality. Unfortunately, there was no information published that described the frequency and the context of unsafe abortion among adolescents in Tanzania.

Limited youth-friendly health services in Tanzania may have an important contribution to the high prevalence of risky sexual behaviors and its consequences. Efforts should be directed to implement service delivery points intended to provide health services more friendly to adolescents so that they are more likely to be able to and willing to obtain the health services they need. Service provider training is also an imperative measure in order to change beliefs and misconceptions of these professionals that hinder the access of adolescents to this service. It is important to increase both health service provision and access among adolescents, helping to adjust knowledge about the problems to the appropriate attitudes, overcoming the distance between, for instance, knowledge about HIV risk factors and condom use.

Education is also a key point that should be taken as a priority. There are different mechanisms by which education could be protective against the acquisition of pregnancy and STIs including HIV. More time in school could lead to higher exposure to sexual and reproductive health education. Accumulating higher levels of education and associated qualifications could improve the young women’s socio-economic position, leaving them less dependent on sexual partners and more empowered to negotiate safer sexual practices such as condom use.

### Limitations

Although this scoping review engaged in a comprehensive search, our inclusion criteria restricted to articles published in English represents a limitation, since it may potentially lead to language bias. Nevertheless, this review delivers a comprehensive overview of the available evidence on sexual and reproductive health behaviors of Tanzanian adolescents, which may contribute to the development of more effective preventive interventions driven to the identified risk behaviors.

## Conclusion

The available published information shows that adolescents engage in high-risk sexual behaviors and experience adverse consequences. Though it seems essential to collect more information, both quantitative and qualitative, the reviewed one evidenced a need for improving the provision of sexual and reproductive youth-friendly health services among Tanzanian adolescents.

## Data Availability

All data generated or analyzed during this study are included in this article.

## Abbreviations

HIV: Human immunodeficiency virus
MDG: Millennium Development Goals
STI: Sexually transmitted infections
